# Ulnar Nerve Dislocation at the Elbow: Review of the Literature and Report of Three Cases

**DOI:** 10.2174/1874325000701010001

**Published:** 2007-09-24

**Authors:** K.C Xarchas, I Psillakis, O Koukou, K.J Kazakos, A Ververidis, D.A Verettas

**Affiliations:** University Orthopaedic Department, Democritus University of Thrace, Alexandroupolis, Greece

**Keywords:** Ulnar nerve dislocation, triceps dislocation, diagnosis, treatment.

## Abstract

Ulnar nerve instability without compression at the cubital tunnel is not common and even more rare is a dislocating nerve. We review the literature regarding the etiology of instability, its incidence and treatment. Snapping around the medial humeral epicondyle can also be caused by a subluxing medial head of the triceps. This pathology may be accompanied by symptoms from the ulnar nerve. Differential diagnosis even intraoperatively is therefore essential if effective treatment is to be given. We also present our own experience on the subject consisting of three cases, one of them with bilateral instability. In only one case there were clinical findings suggesting nerve compression. All laboratory and screening tests were normal, except for the nerve conduction studies in this one case. The main symptom was strong pain, especially during manual activities. Only two of the four subluxing nerves required surgical treatment which in our case was by anterior submuscular or subcutaneous transposition of the ulnar nerve. As diagnosis is not always easy and is usually made on clinical grounds, we also present a clinical test that we believe to be diagnostic for the situation.

## INTRODUCTION

Knowledge of the topographical relationship of the ulnar nerve with the regioned structures is very important. The ulnar nerve arises as the terminal branch of the medial cord of the brachial plexus. At its origin, the ulnar nerve lies medial to the axillary artery and then close to the brachial artery in the anterior compartment of the arm as far as the middle third of the humerus. At this point the ulnar nerve pierces the medial intermuscular fibrous septum to enter the posterior compartment where it lies anterior to the medial head of the triceps muscle. It then passes behind the medial epicondyle to the groove between the olecranon and the medial epicondyle of the humerus. The cubital tunnel begins at the so-called ulnar groove just behind the medial epicondyle of the humerus. It can be divided into three parts: the entrance just posterior to the medial epicondyle, the area of the fascial aponeurosis joining the two heads of the flexor carpi ulnaris, and the muscle bellies of the latter itself. There have been many suggested causes for the entrapment of the ulnar nerve at the cubital tunnel and various surgical techniques can be used to relieve the nerve from pressure [1].

During a time period of six years we have treated three patients with ulnar nerve instability at the elbow.

The term “ulnar nerve instability” describes the chronic conditions of subluxation and relocation of the ulnar nerve at the elbow with flexion and extension of the elbow, respectively. Recurrent subluxation of the nerve at the elbow results in a tractional and frictional neuritis. The nerve is vulnerable to trauma in its subluxed position, lying superficially on the medial humeral epicondyle [2]. This situation is different from the common compression neuropathy.

## CASE REPORTS

**Case 1**. A 33 -year old man, a professional body builder, presented complaining for pain on the medial side of both elbows, especially on the left non - dominant arm. The patient had been symptomatic for one and a half year, his symptoms mainly consisting of inconvenience and a strong pain around the medial humeral epicondyle during manual activities. In the beginning symptoms were provoked by lifting weights or performing push-ups, activities that require a phase of resisted flexion of the elbow beyond 90 to 100 degrees as well as resisted extension. During the last three months symptoms had become worse. The feeling of inconvenience became constant and pain came up even with everyday manual activities. He had therefore had to quit weigh lifting. On examination no clinical findings indicative of nerve compression were found. Tenderness over the medial humeral epicondyle was noted and a possible diagnosis of medial epicondylitis was considered but the picture was not typical. Investigations including plain Xrays, MRI scan and nerve conduction studies were normal. Repeat clinical examinations revealed bilaterally unstable ulnar nerves by palpation. The nerve was felt to sublux anteriorly sliding over the medial epicondyle during flexion and then relocating posteriorly during elbow extension. Though inconvenience and pain were constant the nerve did not appear to dislocate every time the patient flexed and extended his elbow.

**Fig. (1):**
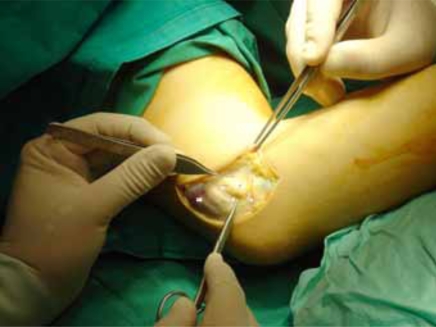
Case 1: The ulnar nerve (UN) immediately after skin incision and initial dissection. At the beginning of flexion the nerve overriding the medial humeral epicondyle (ME) and ready to dislocate anteriorly. FCU: Flexor carpi ulnaris, MHT medial head of triceps.

**Fig. (2):**
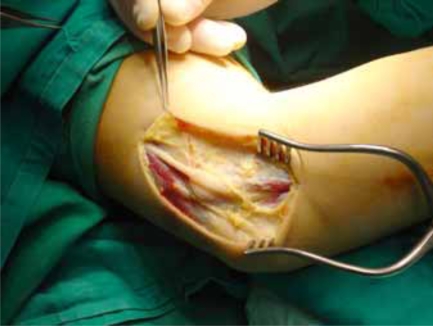
Further dissection.

**Fig. (3):**
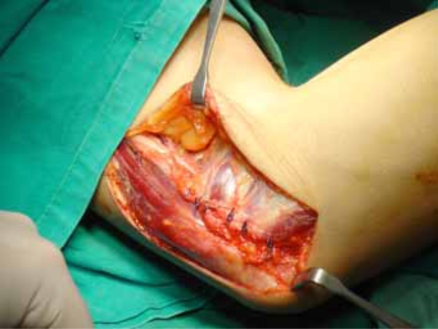
Anterior submuscular transposition of the ulnar nerve completed.

The treatment offered was operative for the non dominant and conservative (strapping, non steroid anti-inflamattory drugs) for the dominant arm. Anterior submuscular transposition of the ulnar nerve combined with minimal epicondylectomy was our surgical treatment of choice (Figs. 1-3). No osseous abnormality such as a hypoplastic medial epicondyle was noted intraoperatively. A long arm back slab was used for three weeks with the elbow in 90^0^ of flexion and a rehabilitation program followed for six more weeks. The patient’s post operative recovery was complication free. Six months postoperatively he was asymptomatic and back to his normal activities including weight lifting. **Case 2**. A 28 year old woman, a waitress by profession presented complaining of a spontaneous snapping around the medial side of her Rt dominant elbow. Occasionally she would feel the snap when lifting a tray while working. She had had the discomfort for about one year when referred to us. All investigations were normal, and there were no signs of ulnar neuropathy. Instability of the ulnar nerve was discovered by palpation during careful clinical examination. Treatment was by conservative means as already described, but the patient who denied surgery had to change her job. **Case 3**. A 35 year old woman, a secretary, presented complaining of symptoms indicating a cubital tunnel syndrome on her Rt dominant elbow. She described that she occasionally felt paresthesiae down her two ulnar fingers when placing her flexed elbow on the desk and trying to type. Nerve conduction studies were positive for ulnar neuropathy at the elbow. Instability of the ulnar nerve was also discovered by palpation during careful clinical examination. Treatment was by anterior subcutaneous transposition of the nerve. Three weeks later she was completely asymptomatic and back to work.

In all cases treatment options were explained to the patients and offered according to the criteria discussed below.

In all three cases we found that the nerve could be stabilized by the examiner’s hand by compression on the medial head of the triceps and lateral displacement. If this was done with the elbow extended, the nerve was stable in its proper position and flexion could then be done without any discomfort. On these grounds we developed a clinical test that we believe to be diagnostic for the situation: The examiner’s fingers and palm are positioned just proximally to the lateral and the thumb about five centimeters proximally to the medial humeral epicondyle. With the patient’s elbow in extension the ulnar nerve is pressed by the thumb to the medial head of the triceps and both pushed laterally while the lesser fingers apply counterpressure. The nerve is thus stabilized behind the medial epicondyle. Continuous flexion – extension of the elbow then performed should not elicit the symptoms as expected by the patient. We believe that this test can be very helpful especially in cases where the nerve is not constantly dislocating and no nerve compression findings are evident.

## DISCUSSION-CONCLUSIONS

Recurring luxation of the ulnar nerve at the elbow is uncommon, occurring about equally in young and old, male and female, athletes and non-athletes but the greater mobility is usually at the dominant arm. The probable cause of such a dislocation is congenital laxity of supporting ligaments. Being more vulnerable to injury than normally positioned nerves, however, complicating neuritis can occur. Subluxating nerves which stop on the tip of the medial humeral epicondyle upon 90 degrees or more of flexion at the elbow, are more subject to direct trauma than completely displaced neural structures which cross the epicondyle upon elbow flexion. The latter may develop friction neuritis which occurs most frequently in industrial workers and occasionally requires surgical transfer [3].

Snapping on the medial side of the elbow, even if it is associated with symptoms related to the ulnar nerve, is not necessarily caused by dislocation of the ulnar nerve alone. Dislocation of the medial head of the triceps can occur in combination with dislocation of the ulnar nerve, producing the clinical finding of at least two snaps at the elbow, with or without discomfort on the medial side of the elbow and with or without ulnar neuropathy. Often snapping of the medial edge of the triceps over the epicondyle is subtle and is detected only with careful palpation. Variations in the anatomy of the triceps have been reported in patients who have recurrent dislocation of the triceps or ulnar neuropathy, or both [4-6]. A prominent medial head of the triceps can be congenital or acquired, whereas an accessory tendon suggests a congenital etiology [7]. Osseous abnormalities on the medial side of the elbow, whether post-traumatic [8] or developmental, also have been associated with dislocation of the triceps, dislocation of the ulnar nerve, or ulnar neuropathy [9-11]. Ulnar nerve instability in patients who have only mild, intermittent, or positional symptoms can usually be managed effectively with non-operative measures, such as avoidance of activities that involve repetitive flexion and extension of the elbow and NSAIDs. While non-operative treatment may decrease symptoms, it usually does not eliminate the underlying snapping. Patients who have persistent painful snapping or symptoms related to the ulnar nerve despite a trial of non-operative therapy should be considered for operative management. There are several surgical alternatives for symptomatic recurrent dislocation of the ulnar nerve. Anterior transposition of the ulnar nerve is usually selected by the majority of orthopaedic surgeons because of the simplicity of the operative procedure and the early return to previous activities [3]. Deep intramuscular implantation, with or without neurolysis, is in our opinion a better option for this situation [1]. Although the natural history of snapping of the triceps is not known, our current treatment of this clinical entity depends on the severity of the symptoms. Snapping that is detected incidentally and painless snapping without symptoms related to the ulnar nerve do not warrant treatment [6]. When symptomatic it should be treated etiologically, usually by lateral transposition or excision of the medial head of the triceps [6,12,13].

Patients who have a transposition of the ulnar nerve, especially those who have dislocation of the ulnar nerve, should be examined intraoperatively with the elbow in flexion and extension so that the surgeon can be certain that the medial head of the triceps does not snap over the medial epicondyle. Failure to recognize concurrent dislocation of the ulnar nerve and the medial head of the triceps can result in persistent, symptomatic snapping after an otherwise successful transposition of the ulnar nerve [6,14].

In our first case instability was bilateral which is far less common and the non-dominant arm was the one requiring surgery, which is also uncommon. The fact that the patient was a weight lifter led us to believe that a hypertrophic medial head of the triceps was the reason of nerve dislocation. It should be mentioned that episodic elbow snapping and ulnar nerve dysesthesias only after weightlifting has also been reported. As symptoms disappear soon afterwards, a minor change in the configuration of the medial portion of the triceps (fluid accumulation) in the same individual at different times is considered to be the causitive factor of intermittent dislocation of the medial triceps [15]. This hypothesis was not confirmed intraoperatively when no triceps snapping was observed during flexion-extension of the elbow. As for the possibility of episodic snapping this could also not be our case because of the constant character of the symptoms. The other two cases were more typical in their presentation, diagnosis and treatment.
